# Hospital Mortuaries and Dead-Houses

**Published:** 1905-08-26

**Authors:** 


### HOSPITAL ADMINISTRATION.

### CONSTRUCTION AND ECONOMICS.

### HOSPITAL MORTUARIES AND DEAD-HOUSES.

THE SCANDALS OF OBLIVION.

Reverence for the dead, and the relations of the living to the dead, are two subjects which in fact are apt to receive far less attention than they merit in these days at the hands of some hospital and anatomy school authorities and the public. It is due to our common humanity that the dead should be treated with respectful decorum, especially when a dead body is in the custody of the authorities of a hospital or a medical school. We are glad to know from personal inspection that the best-administered institutions provide that this essential shall be fulfilled in every mortuary and dead-house. But a recent inspection of the arrangements for cadavers in the dead-houses in connection with certain medical schools in London reveals that abuses have crept in and are allowed to continue, which are opposed to public decency and attended by dangers to the living, which would be incredible but for an absence of knowledge of the evils which result from ignoring an unpleasant subject by silence. For this reason, no doubt, the dead-house or morgue or both of the hospital and medical school has too often been relegated to the limbo of forgotten things, so far as efficient and adequate official inspection is concerned. We refrain from giving details because they must prove appalling to read and shocking to the feeling of decorum which animates the mind of every decent man and woman in this country to-day. We will content ourselves then by directing the attention of the Home Secretary to the urgent need there is for him to immediately order an inspection of the existing morgues and dead-houses attached to the schools of anatomy in London, and to call for a detailed report. It is essential that he should take this step with promptness, because the existing arrangements at some of these morgues are fraught with danger to medical students and teachers, and are wholly opposed to that proper regard and reverence for the dead which should be enforced through the action of the staff of the Home Office and those immediately responsible for the custody of the dead bodies and their preservation during their retention by the schools of anatomy throughout London. On educational grounds, too, it is essential that the Home Secretary should take prompt action, because the dearth of cadavers for educational purposes is likely to continue and increase so long as the existing abominations are allowed to remain in connection with certain of the morgues attached to these anatomical schools in the metropolis.

We have thus far been dealing with the dead-house arrangements of the anatomical schools, but what of the mortuary arrangements of some of the hospitals up and down the country? On Saturday afternoon we happened to be inspecting a large general infirmary supported by voluntary contributions in one of the most important cities in the west of England. When we had finished our inspection we noticed a motor-car at the hospital entrance, and on inquiry found that it had brought in a man whom the motor-car had run over and killed. We were subsequently asked to inspect the casualty department, which consisted of a small room divided by blanket curtains, where 100 casualties and more have to be attended to each day. On the wall to the left of the entrance was a long form, where several patients of both sexes and a child or two were waiting to be seen by the doctor. On lifting the curtain communicating with the portion of the room where the sink and window were, a nurse came from behind a second curtain shutting off a narrow strip at the far side of the room, who intimated that the body brought in by the motor-car was lying there on a couch, and that she was then performing the last offices for the dead. On inquiry we learnt that this couch was practically the only place which the authorities had available for the retention of the dead until the friends came and could make arrangements with an undertaker for the removal of the body.

Do members of the committee never inspect or inquire into the mortuary arrangements and dead-houses?

It seems to us incredible that the authorities of a great general hospital in a wealthy provincial city, which has been established for upwards of 150 years, should have overlooked the imperative necessity for making adequate and decent arrangements for the treatment of its casualties and the reception of accident patients who may expire before they reach the institution. In all our long experience of hospitals and sickness we never remember anything which has surprised or shocked us more than the incident we have just described. It is surely time that every hospital and every school of anatomy throughout the United Kingdom should face the position which we have felt it our duty to call attention to. Whatever other pressing matters there may be to deal with, all else should be set aside until arrangements have been made in every such establishment throughout the country for the decent and hygienic equipment and construction of the mortuaries and dead-houses attached to these institutions.

